# Influence of Phase Composition and Morphology on the Calcium Ion Release of Several Classical and Hybrid Endodontic Cements

**DOI:** 10.3390/ma17225568

**Published:** 2024-11-14

**Authors:** Ivanka Dimitrova, Galia Gentscheva, Ivanka Spassova, Daniela Kovacheva

**Affiliations:** 1 Department of Conservative Dentistry, Faculty of Dentistry, Medical University Sofia, 1 George Sofiiski Str., 1431 Sofia, Bulgaria; ivanka.dimitrova@fdm.mu-sofia.bg; 2 Department of Chemistry and Biochemistry, Medical University-Pleven, 1 St. Kliment Ohridski Str., 5800 Pleven, Bulgaria; galia.gentscheva@mu-pleven.bg; 3 Institute of General and Inorganic Chemistry, Bulgarian Academy of Sciences, Acad. G. Bontchev Str. Bl. 11, 1113 Sofia, Bulgaria; ispasova@svr.igic.bas.bg

**Keywords:** dental calcium-silicate cements (CSCs), mineral trioxide aggregate (MTA), hybrid cements, phase composition, calcium release, PDT

## Abstract

The ability of the cement to release calcium ions, which participate in the remineralization of dentin by forming apatite which improves root canal sealing with time, is of particular importance. Five recently introduced calcium-silicate commercial dental cements were investigated with a view to the influence of the physicochemical characteristics on the possibility of releasing calcium ions in an aqueous medium. Two hybrid calcium-silicate cements in the form of a paste-like ready mix (BioCal^®^ Cap and TheraCal LC) and three calcium-silicate cements consisting of two components—powder and liquid (Harvard MTA Universal, Rootdent, and BioFactor) were subjected to powder XRD, SEM, and EDS for detailed examination. The cements were immersed in water for 28 days and the phase composition and morphology of the cements before and after soaking were studied. The total calcium release for each cement was determined by ICP-OES. BioFactor and BioCal^®^ Cap release the highest amount of calcium ions, while the lowest release is registered with Rootdent and TheraCal LC. The PDT treatment of BioFactor does not influence substantially the calcium release. The impact of the elemental and phase composition on the calcium release and calcium carbonate formation was discussed. A reciprocal relation between the aluminum content and the quantity of the released calcium has been found.

## 1. Introduction

Advances in materials science have promoted the evolution of dental cements and led to the development of different types of cements tailored to specific clinical applications. Historically, dental cements can be categorized into several generations based on their composition and properties, which have evolved to meet the changing demands of restorative dentistry. The earliest cement-like restorative material made of quicklime has been introduced in 1796 by Frederich Hirsch, and was not particularly successful [1]. Furthermore, calcium hydroxide-based materials have been introduced and applied because it has been found that they promote pulp repairing [2]. However, their low mechanical strength and high solubility have led to the search for materials with improved properties [3].

The efforts of scientists have resulted in the introduction of calcium-silicate cements that have shown good clinical results. The era of bioactive calcium-silicate cements (CSCs) in dental practice started in 1993 with the introduction of gray mineral trioxide aggregate (MTA). This first cement was developed by the team of Torabinejad and Dr. White [4] and was immediately embraced with enthusiasm. It has been found to be superior to calcium hydroxide in mechanical indices that makes it suitable for pulp capping [5,6]. In 1999, Pro Root^®^ MTA (Dentsply, Tulsa Dental Specialties, Johnson City, TN, USA) became the first commercially-available CSC in the United States. The dental cement composition is a copy of the composition of the well-known construction Portland cement with the only addition of bismuth oxide (originally) as a radiopaque material [7]. The initially-powdered calcium-silicate Portland cement was set after a hydration reaction with water. The products are different phases of calcium-silicate hydrate (CSH), which are mainly amorphous at the beginning. The crystallization of portlandite (calcium hydroxide), and hydrated calcium aluminates or calcium carbo-aluminates starts after several hours [8,9]. In addition to good sealing ability, calcium-silicate cements demonstrate other important and useful properties mainly related to their biocompatibility and bioactivity, which were demonstrated by strong healing and low inflammatory responses [10,11,12,13]. Calcium-silicate materials provide excellent sealing abilities, with the additional possibilities for stimulating the formation of apatite-like precipitates if reacting with phosphate-containing liquids since they release ions promoting the growth of the dentin bridges [14,15]. Particularly attractive became their ability for “biomimetic remineralization” of dentin, first pointed out by Tay and Pashley [16], which was considered an innovative concept in dentistry and opened the way to potential new applications of this class of materials [17]. The remineralization consists of a non-classic crystallization introduced recently [18,19], where amorphous precursors are transformed into crystalline intermediate phases, which coalesce into microscopic crystals [20].

The hydration reaction concerns the two calcium silicates in the cement according to the following equations:

2Ca_3_SiO_5_  +  6H_2_O⟶Ca_3_Si_2_O_7_.3H_2_O  +  3Ca(OH)_2_ (1)

2Ca_2_SiO_4_ +  4H_2_O⟶Ca_3_Si_2_O_7_.3H_2_O  +  Ca(OH)_2_ (2)

The apatite precipitation reaction proceeds by the following equation:

7Ca(OH)_2_ + 3Ca(H_2_PO_4_)_2_⟶Ca_10_(PO_4_)_6_(OH)_2_ + 12H_2_O (3)

The availability of phosphate ions in the fluid is necessary to form the apatite phase. Various remineralizing mediums may provide phosphate ions, such as simulated body fluid, phosphate-containing solutions or gels, calcium phosphate remineralizing solution, artificial saliva, phosphate-buffered saline, etc. [21]. Calcium phosphate cements simultaneously release calcium and phosphate ions, leading to the direct formation of hydroxyapatite, a mineral that facilitates the regeneration of bone and dental tissues [22,23].

An advantage of CSC materials is that they have an alkaline pH upon setting, thus contributing to the elimination of any remaining harmful microorganisms which promotes the healing of dental pulp [24,25]. The setting reaction of calcium-silicate cements leads to the formation of calcium-silicate hydrate gel, which is responsible for their mechanical strength and bioactivity [26]. Additionally, CSCs are capable of forming a bioactive interfacial layer of calcium phosphate when in contact with dentin, enhancing their bonding to tooth structures [27,28]. Phosphate and silicate dental cements differ in their mechanical properties. Compared to phosphate cements, CSCs generally show superior compressive strength, making them more suitable for applications where high tooth loading is expected [24,29]. The choice between these materials depends on the specific case, with phosphate cements used mainly for applications requiring increased biocompatibility, while silicate cements are preferred for their mechanical strength and sealing capabilities. Over the years, a large number of new materials comprising compositions with, in general, minor modifications of that of the original cement, have been proposed by manufacturers and involved in routine practice [30,31,32,33].

Over time, clinical practice also showed some disadvantages of the initially introduced CSCs such as relatively long setting time, which makes manipulations difficult, staining of tooth structure, low compatibility with other dental materials [34], etc. The recent formulations of CSC materials exhibit a finer grain structure allowing for faster setting, better handling of properties, and reduced staining of the tooth [35,36,37].

Recently, a so-called fourth generation of calcium-silicate materials (resin-modified or hybrid CSC) has been proposed, which combines the classical cement composition with a light-curing resin or monomer component [2,38,39,40]. This provides excellent sealing abilities, with additional possibilities for stimulating apatite-like precipitates and dentin bridges [41,42]. In addition, they offer superior aesthetic performance compared to traditional CSCs [43].

Recent developments in dental cements have focused on enhancing their physical and mechanical properties. As the number of manufacturers increases, the rate of proposing new materials is high and many new products can be expected soon. This is perhaps the reason why the available information and studies on the qualities of new cement brands are scarce. The very important information about their bioactive potential is also insufficient. As mentioned already, the ability to form apatite deposits and dentine bridges is one of the main advantages of these materials which are closely related to the ability to release calcium ions from the cement bodies.

Recently, photodynamic therapy (PDT) demonstrated good antimicrobial potential in root canal system disinfection [44]. The specific pathogenic mixture in the mouth with predominant G-anaerobic bacteria and some fungi is eliminated successfully by PDT [45]. The use of low-power lasers activates successfully an exogenous photosensitizer. The latter produces reactive oxygen species, which are highly toxic to pathogens. The combined treatment of PDT after the use of a direct pulp capping agent or repair of perforation with CSCs raises interest in possible interactions of the materials used for both therapies.

Due to the large number of dental cements offered by various manufacturers, some of them remain far from the attention of researchers, moreover, some of them are new and still not studied. The data in the literature on the morphological characteristics and especially on the changes in the phase composition of the cements during soaking in different fluids are scarce. This work aims to investigate and present new results on the initial phase composition and morphology of five commercial calcium-silicate cements (classical and hybrid types), as well as to study the phase evolution and the morphological changes that occurred after soaking in water to estimate their capability for calcium ions release. The influence of the PDT therapy on the phase composition and morphology of the cement with the best calcium release is assessed.

## 2. Materials and Methods

### 2.1. Samples Investigated

The following recently introduced calcium-silicate dental materials were used in the present study. Their choice is motivated by the intention to compare the cements from different types (hybrid with classical), and to present data for the newly introduced to the market or not well studied (BioFactor, Rootdent).

(1) *Hybrid calcium-silicate cements*

In this case, the manufacturer provides prepared mixtures in a paste-like form in a syringe container:

- TheraCal LC (Bisco Inc., Schaumburg, IL, USA)
- BioCal^®^-Cap (Harvard Dental International, Hoppegarten, Germany)

(2) *Conventional calcium-silicate cements*

The manufacturer provides two components (powder and liquid) for the preparation of these cements with instructions for their proper mixing:

- Harvard MTA Universal (Harvard Dental International, Hoppegarten, Germany)
- Rootdent (TehnoDent, Belgorod, Russia)
- BioFactor (Imicryl, Konya, Turkey)

### 2.2. Preparation Procedure

Silicone molds with a diameter of 15 mm and a depth of 2 mm were used for cement setting. Cements, prepared according to the manufacturer’s instructions were placed in these molds.

For Harvard MTA Universal, the powder/liquid ratio was 2.6/1.0. This can be obtained by mixing 1 level (orange) scoop of powder and 2 drops of liquid. The total mixing time was 30 s.

For the Rootdent, the powder/liquid ratio was 2.8/3.1 and the total mixing time was 1 min.

For BioFactor, the powder/liquid ratio is 1 glass vial of powder and 3 drops of liquid followed by mixing to homogeneous consistency.

Hybrid calcium-silicate cement TheraCal LC was placed in the molds by 1 mm increments and light-cured for 20 s by light curing for polymerization of the resin component with a Bluedent Smart (Plovdiv, Bulgaria) photopolymer lamp, as proposed by the manufacturer.

The same procedure was performed for the other resin-modified cement BioCal^®^-Cap for 40 s.

### 2.3. Photodynamic Therapy Treatment

Photodynamic therapy (PDT) was performed with FotoSan 630 (CMS Dental, Roslev, Denmark) using photosensitizer toluidine blue in low viscosity with a concentration of 0.1 mg/mL. The photosensitizer (0.5 mL) was deposited on the cement surface after the cement had completely hardened. The material was treated with a lamp with a wavelength of 630 nm and an intensity of 2000 mW/cm^2^ twice for 30 s. After the PDT, the sample was washed with 5 mL of distilled water and dried.

Table 1 summarizes the types of the cements used and the corresponding preparation procedures.

### 2.4. Physicochemical Characterization

All materials were analyzed by the powder X-ray diffraction technique. An X-ray diffractometer (Bruker D8-Advance, Carlsruhe, Germany) with CuK_α_ radiation (λ = 1.5418 Å) and Ni filter operating at 40 kV/40 mA mode was used. The patterns were collected with a LynxEye detector in the scan range 5–90°2θ.

The diffraction patterns were evaluated using the EVA V4 software package in combination with the ICDD-PDF-2 database (2021 release).

The elemental composition and the morphology of the cements were investigated using a JEOL-JSM-6390 scanning electron microscope (Tokyo, Japan) equipped with an energy dispersive spectroscopy (EDS) detector (Li, Si).

### 2.5. Calcium Release Experiments

The analyses for the calcium release were performed on ICP-OES iCAP 7000 SERIES (Thermo Fisher Scientific, Waltham, MA, USA) using the filtered solutions obtained after immersing the cements for 28 days in water.

The methodology was as follows: The samples, dried to a constant weight, were weighed on an analytical balance and placed in polypropylene containers, to which deionized water (pH = 6.3) was added in a ratio of 1:20 (*w*/*v*). The containers were closed and placed in a closed incubator at 37 °C for 28 days. The solutions were then filtered through 0.45 μm filters (GVS Group, Bologna, Italy) and the filtrate was analyzed for Ca content. Working solutions with predetermined concentrations were prepared from a standard solution of 1000 mg/L Ca (Merck KGaA, Darmstadt, Germany).

After filtering, the solid mass of each cement is dried and analyzed in the same manner as the initial materials.

## 3. Results

The investigations concerning the influence of the type, phase composition, and morphology of commercial calcium-silicate cements, the phase evolution, and the morphological changes that occurred after soaking in water related to their capability for calcium ions release, led to the following results.

### 3.1. TheraCal LC

**Figure 1:**
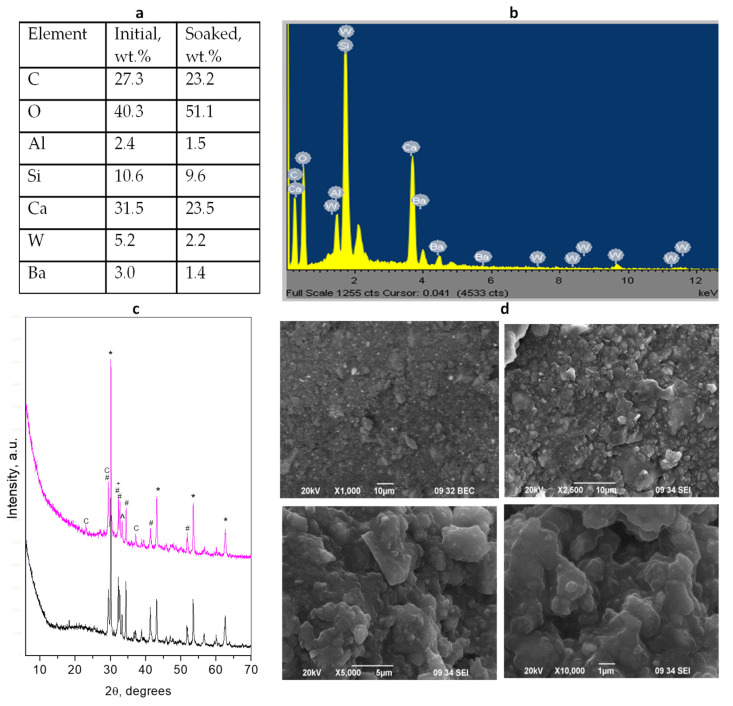
Results of combined analyses of *TheraCal LC* cement: (**a**) Elemental composition of the initial mixture and soaked material; (**b**) EDS spectrum of the initial cement mixture; (**c**) powder diffraction patterns of the initial solidified mixture (black) and after 28 days of water soaking (magenta). Peaks notation *—radiopacifier, #—Ca_3_SiO_5_, ^—Ca_3_Al_2_O_6_, +—Ca_2_SiO_4_, c—calcite; (**d**) SEM images of different magnifications of initial (upper) and soaked (down) cements.

Figure 1 summarizes the results of the physicochemical characterization of the initial solidified cement mixture and the soaked material.

The powder diffraction patterns of the initial hybrid and soaked-in-water contain identical crystalline phases. The crystalline cement phases are found to be Ca_3_SiO_5_ (alite), Ca_2_SiO_4_ (larnite), and Ca_3_Al_2_O_6_. Except for these phases, the soaked-in-water sample shows the presence of a small amount of CaCO_3_ (calcite). An amorphous component is also observed for the soaked material, due to the hydration of silicate phases. A more detailed phase composition is presented in Table S1.

The radiopacifier was identified as the Ba_2_CaWO_6_ phase. The data in the literature on the type of radiopacifier used in this type of material are contradictory. Some papers mentioned it as BaZrO_3_, others—as tungsten-containing compounds [46]. For example, Gandolfi et al. [2] reported that the radiopacifier in this material is a mixture of BaSO_4_ and CaWO_4_. However, EDS analyses do not show the presence of sulfur in our cement sample. These results correlate well with the EDS analyses showing the increase in oxygen content due to the formation of the calcite phase and the hydration.

The photographs of the hybrid TheraCal LC cement represent particles of different sizes with predominantly small grain-like shapes having a mean size of about 1.3 μm [2]. The radiopacifier particles are generally small but tend to aggregate. The resin component is presented as plate-like sheets. The water soaking leads to a change in the morphology, expressed in an edge-smoothing and appearance of particles with a size of about 0.5 μm that are smaller than these of the initial material and large aggregates with a size of about 2 μm. Voids are also presented within this material.

### 3.2. BioCal^®^ Cap

**Figure 2:**
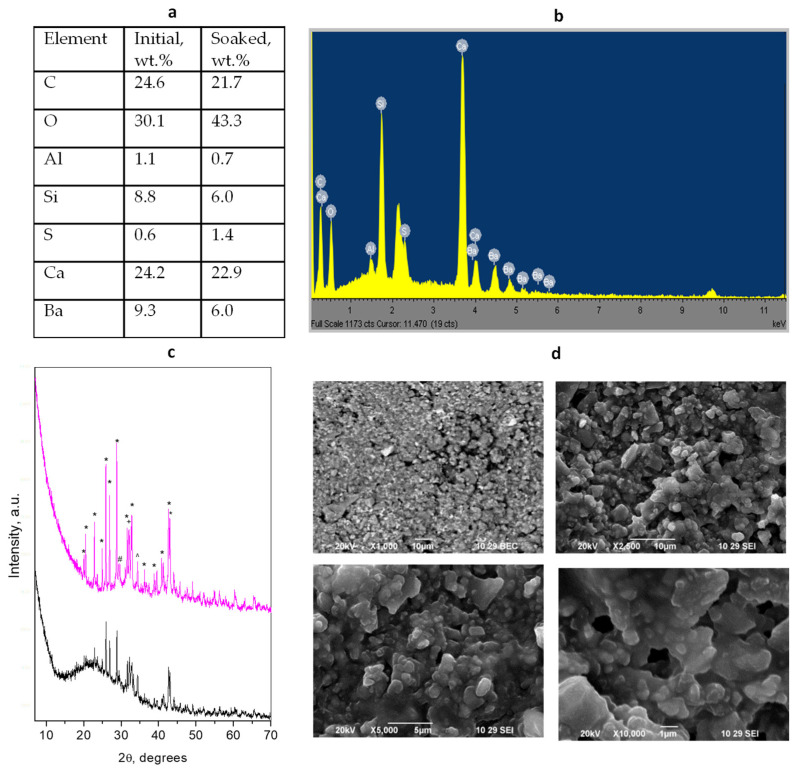
Results of combined analyses of *BioCal^®^ Cap* cement: (**a**) Elemental composition of the initial mixture and soaked material; (**b**) EDS spectrum of the initial cement mixture; (**c**) powder diffraction patterns of the initial solidified mixture (black) and after 28 days of water soaking (magenta). Peaks notation *—radiopacifier, #—Ca_3_SiO_5_, +—Ca_2_SiO_4_, ^—Ca_3_Al_2_O_6_; (**d**) SEM images of different magnifications of initial (upper) and soaked (down) cements.

The XRD patterns of the BioCal^®^ Cap initial hybrid material and soaked-in-water for 28 days are presented in Figure 2. A more detailed phase composition can be found in Table S1. The identified crystalline phases were BaSO_4_ (Barite) as a radiopacifier, as well as the typical cement phases Ca_3_SiO_5_ (alite), Ca_2_SiO_4_ (larnite), and Ca_3_Al_2_O_6_. The resin component of the initial mixture is presented in the form of an amorphous hump in the XRD pattern. Contrary to the TheraCal LC hybrid cement, the increase in the oxygen content is due only to the hydration of the silicate phases as no calcium carbonate phases are observed after soaking.

The SEM images of the BioCal^®^ Cap cement show that the radiopacifier particles are fine and homogeneously distributed in the bulk. The cement particles represent two main fractions with different sizes (0.5 μm and 1.2 μm) which are bound together in a smooth mass of the resin component forming fragments with size varying by about 5–15 μm. For the soaked sample, the resin-grain composite formations (built of 0.5 μm particles) with an average size of about 3 μm are visible with clearly seen 1.5–2 μm voids between them.

### 3.3. Harvard MTA Universal

**Figure 3:**
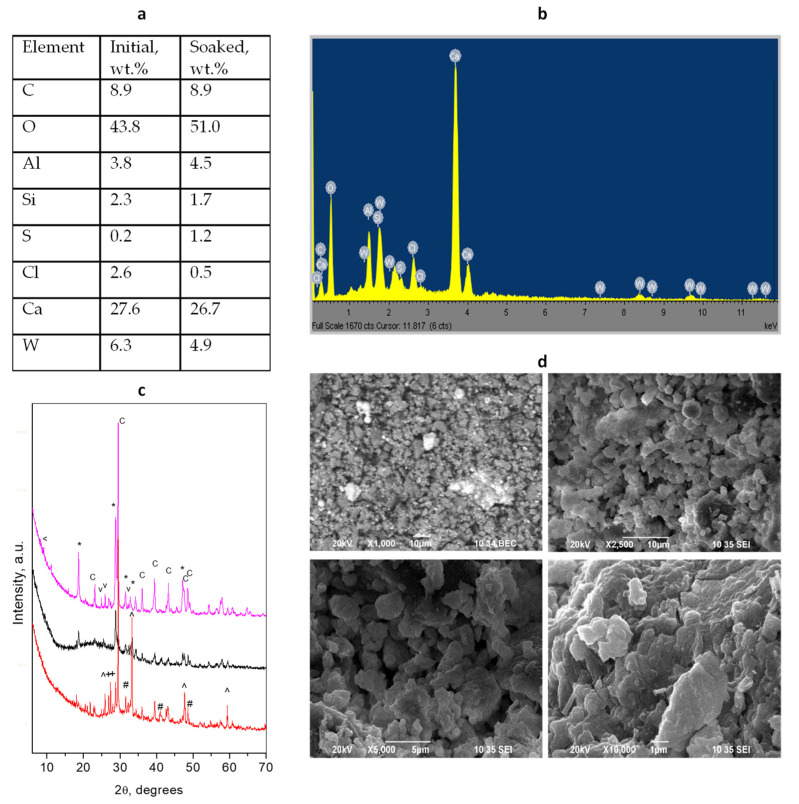
Results of combined analyses of *Harvard MTA Universal* cement: (**a**) Elemental composition of the initial mixture and soaked material; (**b**) EDS spectrum of the initial cement mixture; (**c**) powder diffraction patterns of the initial powder mixture (red), solidified mixture (black), and after 28 days of water soaking (magenta). Peaks notation *—radiopacifier, #—Ca_3_SiO_5_, +—Ca_2_SiO_4_, ^—Ca_3_Al_2_O_6_, c—calcite, v—vaterite, <—hydrated aluminosilicate; (**d**) SEM images of different magnifications of the initial solidified mixture and soaked cement.

Harvard MTA Universal is a representative of the classical type of MTA dental cements. As found by XRD analyses (Figure 3 and Table S1), the initial powder of the Harvard MTA Universal contains CaWO_4_ (scheelite) as a radiopacifier, as well as the typical cement phases like calcium silicates and calcium aluminate. As in the case of TheraCal LC, some authors report other types of radiopacifier [47].

It is worth mentioning that a presence of CaCO_3_ (calcite) in the initial powder was also observed. By mixing this powder with the liquid component, some decrease in the intensities of the peaks of the initial cement crystalline phases occurs and a large amorphous hump is observed originating from the hydration of the cement phases. The radiopacifier and calcite peaks are preserved at this stage. The pattern of the sample soaked in water shows the formation of a new CaCO_3_–vaterite phase and crystalline hydrated alumosilicate. The EDS analyses confirm the increase in the content of calcium carbonate phases and hydrated silicate and alumosilicate phases as an increase in oxygen content is detected after soaking.

The morphology of the initial solidified mixture of Harvard MTA Universal cement (Figure 3) differs from those observed for the hybrid cements TheraCal LC and BioCal^®^ Cap. The cement mass is composed of small well-distinguished (around 1 μm) particles that aggregate in bigger particles with an irregular form (2–5 μm) providing a sufficient amount of small pores between them. The radiopacifier phase is visible as a combination of finely-dispersed small particles, together with the presence of large aggregates. The soaked-in-water sample shows that the initial particles are covered with a layer (probably from a hydrated phase) that joins them together in a denser bulk with a lack of visible voids.

### 3.4. Rootdent

**Figure 4:**
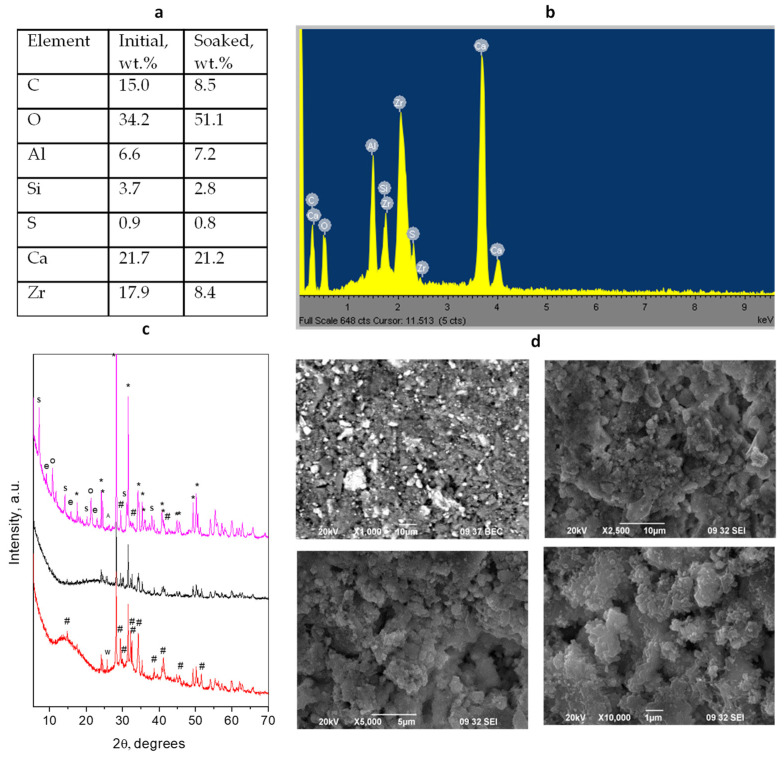
Results of combined analyses of *Rootdent cement*: (**a**) Elemental composition of the initial mixture and soaked material; (**b**) EDS spectrum of initial cement mixture; (**c**) powder diffraction patterns of the initial powder mixture (red), solidified mixture (black) and after 28 days of water soaking (magenta). Peaks notation *—radiopacifier, #—Ca_3_SiO_5_, w—CaAl_4_O_7_, A—aragonite, S—Strätlingite, e—Ca-Al-Si-O, o—Ca-Al-CO_3_-OH-H_2_O; (**d**) SEM images of different magnifications of the initial solidified mixture (upper) and soaked (down) cement.

The initial dry powder for Rootdent material consists of three main phases—ZrO_2_-monoclinic form (baddeleyite) as a radiopacifier also found in [48], Ca_3_SiO_5_ (alite), and calcium aluminate Ca_3_Al_2_O_6_. The presence of an amorphous constituent is also observed as can be seen in Figure 4 and Table S1.

The phase composition of the material being mixed with distilled water is preserved—the main phases being baddeleyite and alite. A significant change occurs after 28 days of immersion of the material in distilled water. An intensive interaction with the water media resulted in several new phases, mainly hydroxides or hydrates, which is evident from the almost twice increase in the oxygen content after soaking. It is worth mentioning that this cement produces a large number of phases after soaking in water. The phases observed in the soaked sample are associated with blended cement hydration like Ca_2_Al_2_SiO_7_.8H_2_O (strätlingite) and some hydroxy carbonates Ca-Al-OH-CO_3_.xH_2_O. These phases are found in cement hydrate systems with high aluminosilicate content [49], consistent with the EDS data for aluminum content.

The particles of the Rootdent cement (Figure 4) look smaller than those of the Harvard MTA Universal (average size 0.5 μm) although they form bigger aggregates (average size 6–7 μm). The radiopacifier particles, clearly seen in the SEM photograph, are relatively large. Pores with irregular form and size are distributed between the cement grains. The soaked sample shows a smoother surface with visible large ~2 μm aggregates made of fine particles (0.2 μm) and voids.

### 3.5. BioFactor

**Figure 5:**
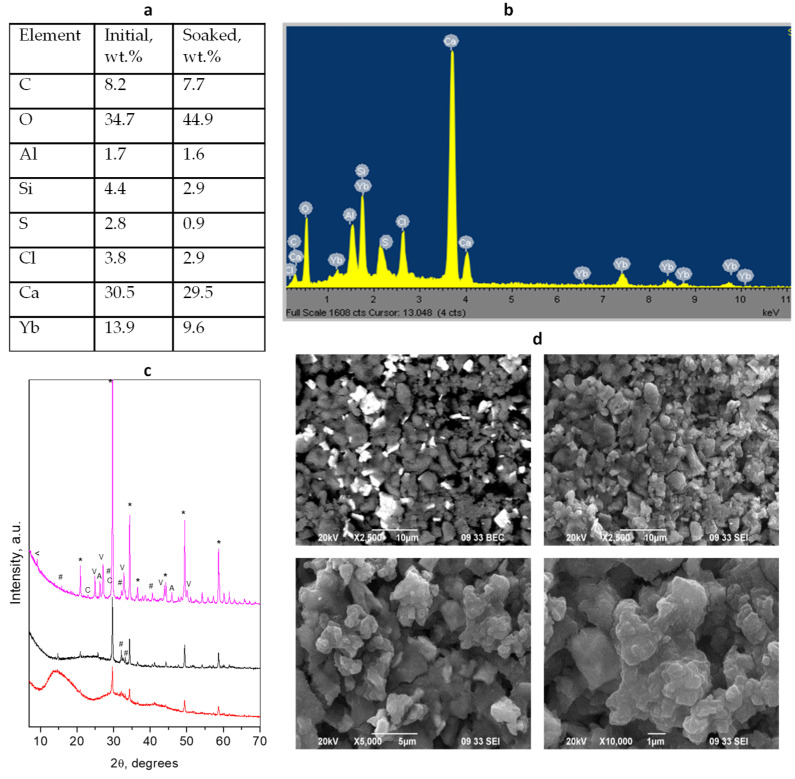
Results of combined analyses of *BioFactor*: (**a**) Elemental composition of the initial mixture and soaked material; (**b**) EDS spectrum of the initial cement mixture; (**c**) powder diffraction patterns of the initial powder mixture (red), solidified mixture (black) and after 28 days of water soaking (magenta). Peaks notation *—radiopacifier, #—Ca_3_SiO_5_, <—hydrated aluminosilicate, c—calcite, A—aragonite, v—vaterite; (**d**) SEM images of different magnifications of the initial solidified mixture (upper) and soaked (down) cement.

A new dental cement BioFactor (Imicryl Dental, Konya, Turkey) has been introduced recently. Figure 5 and Table S1 present the results of the combined analyses on this cement.

The initial BioFactor powder contains an amorphous constituent and peaks of the crystalline Ca_3_SiO_5_ (alite) and radiopacifier Yb_2_O_3_ as also reported in [37]. In the solidified mixture, the same phases are observed together with the disappearance of the amorphous hump. The diffraction pattern of the soaked sample reveals the rise of all three modifications of calcium carbonate (calcite, vaterite, and aragonite). The mass ratio between the content of the three polymorphs is C:V:A = 1:5:3.5. Vaterite polymorph being the least stable polymorph phase of CaCO_3_ in water media easily transforms to a more stable calcite [50]. This transformation is not direct but occurs through dissolution of the vaterite phase in the water and the growth of the calcite phase. Aragonite polymorph of CaCO_3_ is also considered metastable at room temperature. Its formation is supposed to be due to the presence of some external ions (like Na^+^ or Mg^2+^) in the cement mixture [51]. EDS registers the expected increase in the oxygen content.

The SEM images show that the cement comprised large particles with irregular forms with distinct edges. Two fractions (one consisting of big particles ~4 μm, and a second one—of small particles ~1 μm) are clearly distinguished. The Yb_2_O_3_ radiopacifier particles are plate-like and some of them form agglomerates. Upon hydration of the cement, a shell of amorphous hydrated phases of the particles forms, decreasing the differences between the two fractions by connecting them and smoothing the edges of the individual particles.

### 3.6. BioFactor—Photodynamic Therapy (PDT)

**Figure 6:**
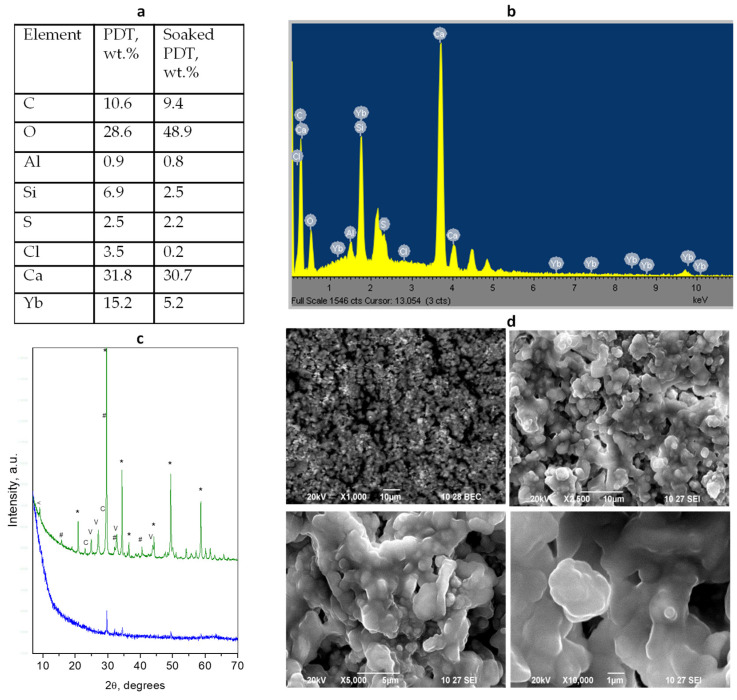
Results of combined analyses of PDT-treated *BioFactor MTA*: (**a**) Elemental composition of the initial mixture and soaked material; (**b**) EDS spectrum of the initial cement mixture; (**c**) powder diffraction patterns of the treated solidified mixture (blue) and after 28 days of water soaking (green). Peaks notation *—radiopacifier, #—Ca_3_SiO_5_, <—hydrated aluminosilicate, c—calcite, v—vaterite; (**d**) SEM images of different magnifications of the PDT-treated solidified mixture (upper) and soaked (down) samples.

Since BioFactor is a relatively new cement in dental practice and there is not much data about it in the literature, we decided to perform an additional study with this material. We studied how the PDT treatment affects the phase transformations in this cement after a long stay in an aqueous environment. The results are presented in Figure 6 and Table S1.

The PDT treatment is made of a solidified cement mixture, with the data presented in Figure 5. The treated sample shows peaks of Yb_2_O_3_ only. An interesting difference between untreated and PDT-treated samples is observed after soaking in water. The secondary formed phases in water for the PDT-treated sample comprise two modifications of calcium carbonate, namely calcite, and vaterite in an approximate mass ratio of 1:4, while in the non-treated sample, all three modifications of CaCO_3_ are observed. The treatment results in a slight change in the elemental composition expressed in the increase in the carbon and oxygen contents, due to the photosensitizer residue.

The PDT treatment affects the morphology of the cement particles expressed in a more pronounced smoothing of the particle edges with an average size of about 1.8 μm and particle coalescing in a common mass with an undefined form. The soaking in water potentiates this transformation leading to the aggregates with a size of about 4–5 μm.

### 3.7. Calcium Release

The release of ions from the cement materials into the dental cavity is mediated by the fluid medium. The ability of the cement to release calcium ions, which participate in the remineralization of dentin by forming apatite which improves root canal sealing with time, is of particular importance [2,52,53,54,55]. The formation of apatite is assisted by the ability of calcium-containing phases to release Ca^2+^ ions in the solution. In this aspect, the most important phases of cements are calcium hydroxide (portlandite) and hydrated calcium silicates [56]. It should be noted that no crystallization of the portlandite phase was observed in the cements included in the present study after immersion in distilled water at 37 °C. This may be due to its low concentration, or to the rapid transformation of this phase into other stable phases as well as to the leaking of calcium ions into the solution.

From this point of view, it is worth studying the possibility of calcium release, especially from newly introduced dental cements, since the data in the literature are scarce.

**Figure 7:**
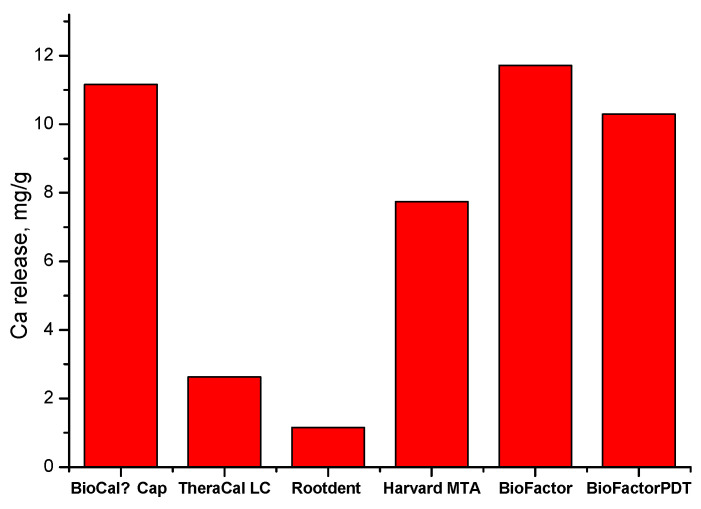
Cumulative Ca release after cement immersion in distilled water at 37 °C for 28 days.

The results as mass of the released calcium per gram cement for the studied materials are presented in Figure 7.

The lowest calcium release is registered for Rootdent cement (1.15 mg/g). The TheraCal LC (2.63 mg/g) and Harvard MTA Universal (7.74 mg/g) cements manifest medium values of evolved calcium ions in the solution, which are comparable to those already reported in the literature [2,57]. On the other hand, the hybrid BioCal^®^-Cap (11.15 mg/g) and the classical BioFactor (11.72 mg/g) show the highest calcium release among the investigated materials. The results do not give us reasons to consider that hybrid materials have an advantage over the classical ones in terms of the calcium ion release indicator. The results also show that the calcium release ability of BioFactor cement after the PDT treatment (10.30 mg/g) is close to the value of the non-treated cement; thus, we may conclude that PDT does not have a substantial effect on the calcium release.

## 4. Discussion

As mentioned in the literature, one of the most important factors for calcium release from cement is the morphology of the particles and their aggregation into a network structure with voids allowing for the diffusion of water through the material. The close examination of the SEM photographs of the initial materials reveals no distinct differences between them. The size of the particles of the initial materials irrespective of their type is between 0.5 and 1.4 μm. The formation of aggregates is observed in both hybrid and conventional cement mixtures, but the size of aggregates varies in a wide range. On the other hand, the soaked materials show rather changed morphology depending on their particular composition. The hybrid cements do not form an overall dense mass (maybe due to the presence of resin component covering part of the cement particles), while for the classic ones the interaction of the cement particles with the water molecules is more facilitated. This observation led to the assumption that phase transformations occurring during the soaking process (registered by XRD analyses) influence the final morphology of the cement. In this context, the phase composition appears to play a crucial role in the calcium release ability.

The hydration of cement proceeds through a complex combination of chemical processes depending on the various constituents in the cement matrix. The rate of hydration of components presented in the particular mixture is important for the final structure, morphology, and properties of the corresponding cement [58,59].

It is well known that the calcium aluminate phase is the most reactive of the main cement constituents, and it reacts first with water to form an aluminate-rich gel. This gel could cover some of the calcium-silicate particles thus leaving them capsulated preventing their reaction with water. As a result, the peaks of Ca_3_SiO_5_ are well seen in the diffraction patterns of the initial mixtures. After soaking, the intensities of the peaks of silicate phases decrease and peaks of hydrated phases and calcium carbonates are observed. The results advocate that the higher aluminum content alters the silicate hydration reactions, potentially affecting the rate of calcium ions release.

The comparison of the results presented in Figure 7 with the data for the elemental composition of the studied cements confirms that the higher calcium release is generally registered by cements with the lower aluminum content.

An interesting result of the present investigation is the absence of portlandite in all the samples and the variety of the calcium carbonate polymorphs registered in the XRD analyses of the cements under study. BioCal^®^ Cap does not contain a calcium carbonate phase, neither in the initial mixture nor in the soaked sample. Within the initially-settled cement mixtures, only Harvard MTA shows the presence of calcite.

On the other hand, the soaked samples show the presence of calcium carbonate in the form of calcite for TheraCal LC and calcite and vaterite for Harvard MTA Universal. Aragonite and traces of calcite are found for Rootdent. The soaked BioFactor contains all three polymorphs, while the PDT-treated BioFactor contains only vaterite and calcite. Among the calcium carbonate polymorphs, calcite is the most stable, aragonite is stable at high pressure and metastable at ambient conditions. Vaterite is the least stable form of CaCO_3_ [42]. It is formed as an intermediate phase during the process of transition of the amorphous state of CaCO_3_ to a more stable calcite/aragonite [60,61]. This process occurs from minutes to hours and the type of the resulting phase depends on the physical and chemical conditions (temperature, pressure, presence of alien ions). Vaterite polymorph can be found in many biogenic systems. It has high porosity and high solubility in water that makes it a suitable material for bone tissue applications and biomedical engineering [62,63]. The solubility of the CaCO_3_ polymorphs increases in the sequence: calcite < aragonite < vaterite, leading to calcite formation at any temperature, and the growth of calcite crystals takes place through dissolution of the metastable form of CaCO_3_.

As our experiments for the calcium release in water are conducted at the same temperature (37 °C), we suggest that the observed variations in the calcium carbonate polymorphs are induced by the presence of alien ions. For example, in the hybrid cements (BioCal^®^ Cap, TheraCal LC), CaCO_3_ exists only in the form of calcite. The metastable forms of CaCO_3_ are observed after soaking all classical cements under study. The transition to calcite occurs via vaterite for Harvard MTA Universal and PDT-treated BioFactor, while it occurs via aragonite for Rootdent. For the non-treated BioFactor cement, the most complex phase transition occurs during soaking, which might be connected to the higher calcium release from it.

## 5. Conclusions

Five recently-introduced calcium-silicate dental cements are investigated to evaluate the influence of some of their physicochemical characteristics on their ability to release calcium ions in aqueous media, considering the importance of the latter in stimulating biomimetic remineralization of teeth. It has been found that BioFactor and BioCal^®^ Cap release the highest amount of calcium ions, while the lowest release is registered with Rootdent and TheraCal LC. In this aspect, hybrid cements do not show clear advantages over classic compositions. The PDT treatment of BioFactor does not influence substantially the calcium release. All investigated cements show a similar morphology before soaking in water. After 28 days of soaking, the morphology changes depending on the specific composition of the cement. A reciprocal relation between the aluminum content and the quantity of the released calcium has been found. The release of calcium ions and the formation of calcium carbonate in aqueous media are related processes, and studying the crystallization mode of calcium carbonate would provide important information for new biomineralization models and the development of new dental materials. The results also may serve as a basis for further investigation on the long-term stability and the biocompatibility of these cements in clinical applications.

## Figures and Tables

**Table 1:** Cements used and preparation procedures.

Cement Used	Manufacturer	Type	Consistency	Preparation
TheraCal LC	Bisco Inc., Schaumburg, IL, USA	hybrid	paste-like form in a syringe container	placed in the molds by 1 mm increments and light-cured for 20 s with a photopolymer lamp
BioCal^®^-Cap	Harvard Dental International, Germany	hybrid	paste-like form in a syringe container	placed in the molds by 1 mm increments and light-cured for 40 s with a photopolymer lamp
Harvard MTA Universal	Harvard Dental International, Germany	classic	powder/liquid	p/l ratio—2.6/1.0 total mixing time 30 s
Rootdent	TehnoDent, Russia	classic	powder/liquid	p/l ratio—2.8/3.1 total mixing time 1 min
BioFactor	Imicryl, Turkey	classic	powder/liquid	p/l ratio—1 glass vial/3 drops mixing to homogeneous consistency
BioFactor PDT	Imicryl, Turkey	classic	powder/liquid	p/l ratio—1 glass vial/3 drops mixing to homogeneous consistency PDT-toluidine blue 0.1 mg/mL wavelength 630 nm, intensity of 2000 mW/cm^2^ twice for 30 s washed with 5 mL of distilled water and dried

## Data Availability

Data are contained within the article and Supplementary Materials.

## Supplementary Materials

The following supporting information can be downloaded at: https://www.mdpi.com/article/10.3390/ma17225568/s1, Table S1. Phase composition according to XRD analyses.
