# Supplementary material for: Influence of Phase Composition and Morphology on the Calcium Ion Release of Several Classical and Hybrid Endodontic Cements
Source: Materials (Basel). 2024 Nov 14;17(22):5568. doi: 10.3390/ma17225568 (PMC11595929; doi:10.3390/ma17225568)
Supplement: Supplementary file 1 [file materials-17-05568-s001.zip › materials-3293200-supplementary.pdf]

Supplementary Materials

Influence of phase composition and morphology on the calcium ion release of several classical and hybrid endodontic cements

Ivanka Dimitrova[1], Galia Gentscheva[2, 3], Ivanka Spassova[3], Daniela Kovacheva[3*]

Table S1. Phase composition according to XRD analyses

Cement	Main phase	Main phase	Main phase	Main phase	Radiopacifier	Trace phases
**TheraCal LC**	Ca_3SiO_5 ICDD-PDF2#00-049-0442 (Main peaks at 2θ - 29.23, 32.06, 32.38)		$Ca_3Al_2O_6$ ICDD-PDF2#00-038-1429 (Main peaks at 2θ - 33.09, 47.33, 59.24)	Ca_2SiO_4 ICDD-PDF2#00-061-0379 (Main peaks at 2θ - 25.71, 31.80, 33.02)	Ba_2CaWO_6 ICDD-PDF2#01-073-0136 (Main peaks at 2θ - 30.08, 43.11, 53.34)	
**Soaked TheraCal LC**	Ca_3SiO_5 ICDD-PDF2#00-049-0442 (Main peaks at 2θ - 29.23, 32.06, 32.38)	C-$CaCO_3$ ICDD-PDF2#00-024-0027 (Main peaks at 2θ - 22.94, 29.49, 39.43)	$Ca_3Al_2O_6$ ICDD-PDF2#00-038-1429 (Main peaks at 2θ - 33.09, 47.33, 59.24)		Ba_2CaWO_6 ICDD-PDF2#01-073-0136 (Main peaks at 2θ - 30.08, 43.11, 53.34)	Ca-Al-Si-O ICDD-PDF2#00-014-0081
**BioCal® Cap**	Ca_3SiO_5 ICDD-PDF2#00-049-0442 (Main peaks at 2θ - 29.23, 32.06, 32.38)		$Ca_3Al_2O_6$ ICDD-PDF2#00-038-1429 (Main peaks at 2θ - 33.09, 47.33, 59.24)	Ca_2SiO_4 ICDD-PDF2#00-061-0379 (Main peaks at 2θ - 25.71, 31.80, 33.02)	$BaSO_4$ ICDD-PDF2#01-070-7037 (Main peaks at 2θ - 25.80, 26.96, 28.80)	
**Soaked BioCal® Cap**	Ca_3SiO_5			Ca_2SiO_4	$BaSO_4$	

	ICDD-PDF2#00-049-0442<br>(Main peaks at 2θ - 29.23, 32.06, 32.38)			ICDD-PDF2#00-061-0379<br>(Main peaks at 2θ - 25.71, 31.80, 33.02)	ICDD-PDF2#01-070-7037<br>(Main peaks at 2θ - 25.80, 26.96, 28.80)	
**Harvard MTA Universal initial powder mixture**	Ca_3SiO_5<br>ICDD-PDF2#00-049-0442<br>(Main peaks at 2θ - 29.23, 32.06, 32.38)			Ca_2SiO_4<br>ICDD-PDF2#00-061-0379<br>(Main peaks at 2θ - 25.71, 31.80, 33.02)	$CaWO_4$<br>ICDD-PDF2#01-071-6152<br>(Main peaks at 2θ - 18.64, 28.77, 31.40)	
**Harvard MTA Universal solidified mixture**	Ca_3SiO_5<br>ICDD-PDF2#00-049-0442<br>(Main peaks at 2θ - 29.23, 32.06, 32.38)	C-$CaCO_3$<br>ICDD-PDF2#00-024-0027<br>(Main peaks at 2θ - 22.94, 29.49, 39.43)	$Ca_3Al_2O_6$<br>ICDD-PDF2#00-038-1429<br>(Main peaks at 2θ - 33.09, 47.33, 59.24)		$CaWO_4$<br>ICDD-PDF2#01-071-6152<br>(Main peaks at 2θ - 18.64, 28.77, 31.40)	
**Soaked Harvard MTA Universal**		C-$CaCO_3$<br>ICDD-PDF2#00-024-0027<br>(Main peaks at 2θ - 22.94, 29.49, 39.43)<br>V-$CaCO_3$<br>ICDD-PDF2#00-024-0030<br>(Main peaks at 2θ - 24.91, 27.04, 32.66)			$CaWO_4$<br>ICDD-PDF2#01-071-6152<br>(Main peaks at 2θ - 18.64, 28.77, 31.40)	Ca-Al-Si-O<br>ICDD-PDF2#00-014-0081<br>$Ca_2Al_3Si_3O_{12}(OH)$
**Rootdent initial powder mixture**	Ca_3SiO_5<br>ICDD-PDF2#00-049-0442<br>(Main peaks at 2θ - 29.23, 32.06, 32.38)				ZrO_2<br>ICDD-PDF2#00-024-1165<br>(Main peaks at 2θ - 24.01, 28.22, 31.45)	amorphous
**Rootdent**	Ca_3SiO_5				ZrO_2	

**solidified mixture**	ICDD-PDF2#00-049-0442<br>(Main peaks at 2θ - 29.23, 32.06, 32.38)				ICDD-PDF2#00-024-1165<br>(Main peaks at 2θ - 24.01, 28.22, 31.45)	
**Soaked Rootdent**	Ca_3SiO_5<br>ICDD-PDF2#00-049-0442<br>(Main peaks at 2θ - 29.23, 32.06, 32.38)	A-$CaCO_3$<br>ICDD-PDF2#00-001-0628<br>(Main peaks at 2θ - 26.16, 33.15, 45.82)			ZrO_2<br>ICDD-PDF2#00-024-1165<br>(Main peaks at 2θ - 24.01, 28.22, 31.45)	Ca-Al-Si-O<br>ICDD-PDF2#00-014-0081<br>$Ca_2Al_2SiO_7.8H_2O$<br>$Ca_4Al_2O_6(CO_3)_{0.5}$ (OH).11.5 H_2O<br>$Ca_4Al_2O_6CO_311H_2O$
**BioFactor**	Ca_3SiO_5<br>ICDD-PDF2#00-049-0442<br>(Main peaks at 2θ - 29.23, 32.06, 32.38)		$Ca_3Al_2O_6$<br>ICDD-PDF2#00-038-1429<br>(Main peaks at 2θ - 33.09, 47.33, 59.24)	Ca_2SiO_4<br>ICDD-PDF2#00-061-0379<br>(Main peaks at 2θ - 25.71, 31.80, 33.02)	Yb_2O_3<br>ICDD-PDF2#01-072-8494<br>(Main peaks at 2θ - 29.66, 34.36, 49.37)	
**Soaked BioFactor**		V-$CaCO_3$<br>ICDD-PDF2#00-024-0030<br>(Main peaks at 2θ - 24.91, 27.04, 32.66)<br>A-$CaCO_3$<br>ICDD-PDF2#00-001-0628<br>(Main peaks at 2θ - 26.16, 33.15, 45.82)<br>C-$CaCO_3$<br>ICDD-PDF2#00-024-0027<br>(Main peaks at 2θ - 22.94, 29.49, 39.43)			Yb_2O_3<br>ICDD-PDF2#01-072-8494<br>(Main peaks at 2θ - 29.66, 34.36, 49.37)	Ca-Al-Si-O<br>ICDD-PDF2#00-014-0081

**BioFactor (PDT)**	Ca_3SiO_5<br>ICDD-PDF2#00-049-0442<br>(Main peaks at 2θ - 29.23, 32.06, 32.38)				Yb_2O_3<br>ICDD-PDF2#01-072-8494<br>(Main peaks at 2θ - 29.66, 34.36, 49.37)	
**Soaked BioFactor (PDT)**		V-$CaCO_3$<br>ICDD-PDF2#00-024-0030<br>(Main peaks at 2θ - 24.91, 27.04, 32.66)<br>C-$CaCO_3$<br>ICDD-PDF2#00-024-0027<br>(Main peaks at 2θ - 22.94, 29.49, 39.43)			Yb_2O_3<br>ICDD-PDF2#01-072-8494<br>(Main peaks at 2θ - 29.66, 34.36, 49.37)	Ca-Al-Si-O<br>ICDD-PDF2#00-014-0081
